# Diagnostic Challenges in Ovarian Hyperthecosis: Clinical Presentation with Subdiagnostic Testosterone Levels

**DOI:** 10.1155/2022/9998807

**Published:** 2022-01-18

**Authors:** Sanket Shah, Callie Torres, Naser Gharaibeh

**Affiliations:** ^1^Department of Internal Medicine, Medical College of Wisconsin, Milwaukee, WI 53226, USA; ^2^Department of Pathology & Immunology, Washington University in St Louis, St Louis, MO 63110, USA; ^3^Department of Endocrinology, Mercy Hospital, 100 Mercy Way, Joplin, MO 64804, USA

## Abstract

Symptoms of hyperandrogenism and virilization in postmenopausal women warrant workup for ovarian hyperthecosis. In this case series, we discuss two patients who presented with symptoms of hyperandrogenism and metabolic abnormalities including insulin resistance stemming from ovarian hyperthecosis. Imaging revealed normal ovaries in both patients. However, both patients had total serum testosterone levels below the lower diagnostic limit for ovarian hyperthecosis. Due to high clinical suspicion of ovarian hyperthecosis, both patients underwent bilateral oophorectomy without venous sampling for ovarian androgens. The diagnosis of ovarian hyperthecosis was confirmed on histological examination. Both women had improvement in their hyperandrogenic symptoms, testosterone levels, and biochemical features of insulin resistance after surgical intervention. This presentation of ovarian hyperthecosis with subdiagnostic total serum testosterone levels demonstrates the need for continued research into the pathophysiology of the disease, discussion of the diagnostic threshold of total serum testosterone, as well as the inclusion of ovarian hyperthecosis in the differential of postmenopausal women with hyperandrogenism and insulin resistance.

## 1. Introduction 

Ovarian hyperthecosis refers to a process in which ovarian cells differentiate into clusters of luteinized thecal cells in the ovarian stroma producing excessive amounts of testosterone. This condition commonly presents in postmenopausal women with symptoms similar to that of polycystic ovarian syndrome [1]. The disorder can result in metabolic abnormalities such as insulin resistance which can then lead to hyperinsulinemia. Hyperinsulinemia can result in an increased risk for type 2 diabetes mellitus and cardiovascular disease [2]. Acne and hirsutism secondary to high testosterone levels are also commonly present [3]. In this article, we discuss two patients with symptomatic ovarian hyperthecosis and laboratory levels below the standard diagnostic criteria. The discussed cases highlight the importance of using clinical impression and metabolic symptomology to confirm the diagnosis of ovarian hyperthecosis, although testosterone levels may be below the usual diagnostic threshold for the disease [4].

## 2. Case Presentation

### 2.1. Case 1

A 60-year-old female with a history of dyslipidemia, hypertension, polycystic ovarian syndrome, and obstructive sleep apnea was referred to endocrinology for management of severe hirsutism, fatty liver disease, hyperlipidemia, and type 2 diabetes mellitus. She reported a longstanding history of hirsutism with frontal balding that progressed over five years and was being treated with spironolactone. She described her libido as normal and denied any clitoromegaly or acne. Compared to one year prior, laboratory results revealed mild elevations in ALT (increasing from 34 to 52) and AST (increasing from 34 to 43). Additionally, hemoglobin A1c (HbA1c) increased from 6.7% to 8.0%. Total serum testosterone levels were at 145 ng/dL.

The patient was started on metformin 2000 mg and subcutaneous semaglutide 0.5 mg to manage her diabetes. Due to her abnormal liver enzyme levels, she was screened for chronic hepatitis which was negative. Pelvic ultrasound revealed a 2.5 cm simple cyst within the right ovary and an incidental 9 mm calcification within the endometrial canal. Her gonadotropin levels were below normal for her postmenopausal state, and DHEA-S, 17-hydroxyprogesterone, and prolactin levels were normal. She was referred to an obstetrician-gynecologist due to suspicion of ovarian hyperthecosis. No adrenal/ovarian vein sampling was performed as it was not available at the facility.

**Figure 1:**
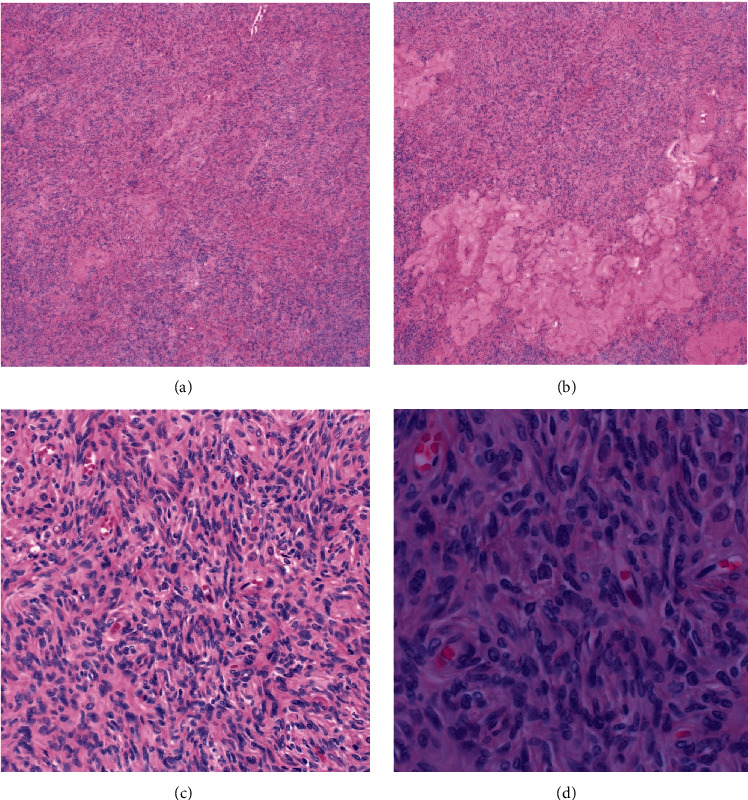
Microscopic images depict ovarian hyperthecosis with stromal hyperplasia and abundant spindled theca cells. Corpora albicantia (B) was also apparent throughout the specimen. Hematoxylin and eosin stain, original magnification: (a) 4x, (b) 4x, (c) 20x, and (d) 40x.

After discussion with the patient and the gynecological team, it was decided that a complete hysterectomy was a reasonable option due to the high suspicion of ovarian hyperthecosis and the presence of rectocele and cystocele. Posthysterectomy, the pathology report was remarkable for atrophic endometrium and bilateral ovarian hyperthecosis as shown in Figure 1. Labs collected during the one-month posthysterectomy follow-up visit were significant, in which liver transaminase levels had returned to normal and HbA1c had improved to 6.3%. Additionally, total serum testosterone decreased to 7 ng/dL, and the patient noted that her hirsutism had mildly improved.

### 2.2. Case 2

A 58-year-old female with a history of hysterectomy in 1999 and hyperlipidemia was referred to endocrinology for management of type 2 diabetes mellitus and hirsutism. She noted excessive hair growth for the past three years which required her to shave and pluck her facial hair daily. She also complained of worsening acne, folliculitis, clitoromegaly, and occasional light vaginal bleeding. She described her libido as normal. She voiced concerns about a rising HbA1c increasing from normal to 8.2% over a six-month period. Additional laboratory tests showed a normal TSH at 3.38 uU/mL and elevated total serum testosterone of 135 ng/dL.

The patient was started on metformin 2000 mg for management of her diabetes. The patient's rapid progression of diabetes and recent development of hirsutism was worrisome for insulin resistance in the setting of an underlying etiology such as high testosterone levels. Pelvic ultrasound showed normal bilateral ovaries containing small cysts or follicles. Pelvic MRI demonstrated bilateral ovarian enlargement for the patient's age, which was more pronounced in the right ovary than the left ovary. Additional laboratory testing revealed normal prolactin, 17-hydroxyprogesterone, and androstenedione levels. However, the patient had subnormal age-specific LH and FSH levels. Based on the imaging and laboratory results, the patient was referred to the gynecological team. The patient then underwent bilateral oophorectomy due to a high suspicion of ovarian hyperthecosis.

**Figure 2:**
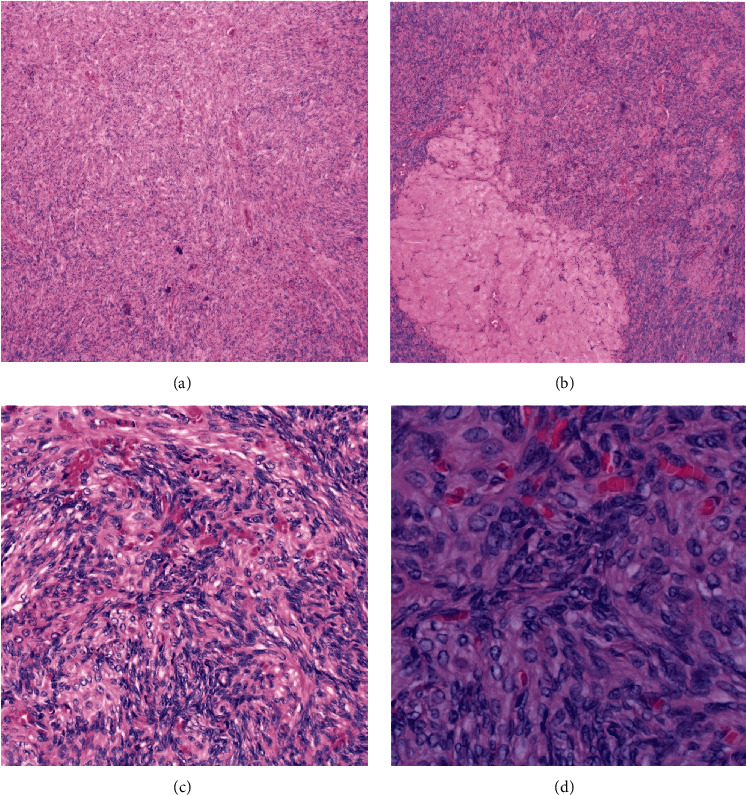
Microscopic images depicting ovarian hyperthecosis with stromal hyperplasia and abundant spindled theca cells. Corpora albicantia (B) was also apparent throughout the specimen. Hematoxylin and eosin stain, original magnification: (a) 4x, (b) 4x, (c) 20x, and (d) 40x.

The oophorectomy pathology report was significant for bilateral ovarian hyperthecosis as shown in Figure 2. Laboratory results collected one-month postoophorectomy demonstrated an improvement in HbA1c from 8.2% to 6.1% and testosterone from 135 ng/dL to 16 ng/dL. The patient denied significant improvement in hirsutism after the surgery and stated that she continued to have excess hair on the abdomen and bilateral upper extremities. However, she reported significant improvement in clitoromegaly.

## 3. Discussion

These cases emphasize the importance of including ovarian hyperthecosis in the differential diagnosis when a postmenopausal woman presents with hirsutism, symptoms of virilization, or symptoms of rapidly progressive insulin resistance (in the absence of clinical or biochemical abnormalities of other etiologies such as Cushing's syndrome or hypothyroidism). Current guidelines recommend that ovarian hyperthecosis is less likely if total serum testosterone levels are below 150 ng/dL [4]. As described in the cases, both patients had a total serum testosterone level below 150 ng/dL and were still diagnosed with ovarian hyperthecosis and had significant improvement in symptoms postoophorectomy. In patients with symptoms of severe hyperandrogenism, it is important to rule out androgen-secreting ovarian and adrenal tumors. In the cases discussed, adrenal tumors were lower on the differential due to normal adrenal androgen levels. A gonadotropin secreting pituitary adenoma was also unlikely given that the patients did not have any suggestive symptoms or laboratory findings consistent with adenoma. Congenital adrenal hyperplasia was additionally ruled out due to normal 17-hydroxyprogesterone levels. Exposure to synthetic androgens was also ruled out due to normal androstenedione levels and patient denial of exogenous hormone intake. Both patients had a significant improvement in their hemoglobin A1C as well as their total serum testosterone levels after oophorectomy as given in Table 1.

In the workup of patients presenting with elevated total serum testosterone levels and virilization, the first step is to identify the source of the excess androgen production. Pelvic ultrasound in women with hyperthecosis is recommended to rule out androgen-producing tumors and usually shows a bilateral increase in ovarian size and stroma [5]. If imaging is negative and a strong suspicion of ovarian hyperthecosis remains, the next step is to perform ovarian venous sampling. It is important to note, however, that ovarian venous sampling is an advanced procedure requiring technical expertise, and evidence for using it in clinical practice is not substantiated [6, 7]. Additionally, the procedure is not offered at all facilities.

Insulin resistance is a key component in identifying ovarian hyperthecosis [8]. It is important to measure sex hormone-binding globulin (SHBG) levels as low levels can serve as a predictive marker for insulin resistance when underlying hyperandrogenism is a suspected cause [9]. Once hyperthecosis is suspected, it is vital to address and manage the various complications caused by increased androgen levels. Patients should be counseled on weight control, lifestyle modifications, and started on metformin for better glycemic control if HbA1c is elevated [10]. The definitive treatment for patients with hyperthecosis is bilateral oophorectomy, and diagnosis of hyperthecosis is confirmed only by histological analysis. If patients are unable to tolerate surgery, treatment with a long-term gonadotropin-releasing hormone agonist is recommended to suppress the HPG axis. Currently, a surgical approach is preferred for the management of hyperthecosis over medical management [3, 11].

## 4. Conclusion

In postmenopausal patients presenting with symptoms of hyperandrogenism, it is important to evaluate for malignant androgen-producing ovarian or adrenal tumors, congenital adrenal hyperplasia, and Cushing's disease. While the testosterone levels in patients discussed were not markedly elevated, the clinical picture was suggestive of ovarian hyperthecosis. After reviewing the patient history, physical exam findings, imaging, and laboratory results, the diagnosis of hyperthecosis was at the top of the differential. Postoophorectomy, both patients showed remarkable improvement in HbA1c and serum testosterone levels. Interestingly, the identification and diagnosis of ovarian hyperthecosis in the presented cases involved testosterone levels below the recommended diagnostic threshold. The discussed cases show that there is a need for continued research and a further discussion into the diagnostic guidelines to better understand the pathophysiology of this disease including the clinical presentation and biochemical findings.

## Figures and Tables

**Table 1:** Pre and postbilateral oophorectomy laboratory results for case 1 and case 2.

	Case 1: pre and postsurgery lab results		Case 2: pre and postsurgery lab results	
Labs	Presurgery	Postsurgery	Presurgery	Postsurgery
HbA1c (%)	8	6.3	8.2	6.1
Serum testosterone (ng/dL)	145	7	135	16
LH (IU/L)	15.9	N/A	21.2	49.9
FSH (IU/L)	20.9	24.4	31.5	74.1
DHEA-S (mcg/dL)	25		47	
17-Hydroxyprogesterone (ng/dL)	72		195	
Prolactin (ng/mL)	8.8		7.9	
Hb (g/dL)	14.0	12.7	14.7	14.8
Hct (%)	42.3	41.0	45.5	46.2

## Data Availability

No data were used to support this study.
